# Association mapping of hybrid seed set and heterosis in a central European multi-parental wheat population

**DOI:** 10.1007/s00122-026-05247-0

**Published:** 2026-04-28

**Authors:** Sandra L. Zapata, Vilson Mirdita, Philipp H. G. Boeven, Pierrick Varenne, Monika Spiller, Shobhashree Nagireddy, Mario Gils, Jochen C. Reif, Guoliang Li

**Affiliations:** 1 Leibniz Institute of Plant Genetics and Crop Plant Research (IPK) Gatersleben, Seeland, Germany; 2 Limagrain GmbH, Salderstr. 4, 31226 Peine-Rosenthal, Germany; 3 Limagrain Europe, Ferme de L’Etang – BP3, 77390 Verneuil L’Etang, France; 4 KWS Lochow GmbH, Wetze 3, Northeim, Germany; 5 Saatzucht Bauer GmbH, Bernburg, Germany; 6 Nordsaat Saatzucht GmbH, Langenstein, Germany

## Abstract

**Key Message:**

Heterosis in wheat results from a complex interplay of dominance and epistasis, with epistasis playing a major role, underscoring the genetic complexity and challenges in systematically exploiting hybrid vigor.

**Abstract:**

The systematic exploitation of heterosis has the potential to increase wheat yields by around 10% compared to inbred lines. However, the lack of natural cross-pollination in wheat significantly increases the cost of producing hybrid seeds. Our study aimed to address these issues by identifying indirect traits that enhance pollination efficiency in hybrid seed production and by genetically dissecting heterosis for key agronomic traits in the resulting hybrids. We evaluated the pollen donor capacity of 294 doubled haploid (DH) lines derived from ten biparental populations adapted to Central European conditions. While no universally applicable indirect predictors of pollination ability were identified, we found that population-specific traits correlated with pollinator performance. We evaluated the resulting hybrids and their parental lines in multi-location field trials for grain yield, plant height, heading date, and susceptibility to yellow and leaf rust. Quantitative trait locus (QTL) analysis revealed that heterosis in wheat is governed by a complex interplay of dominance effects and epistatic interactions. The relative contribution of these genetic effects varies significantly across traits and populations. Epistasis notably played a substantial role in heterosis, emphasizing the genetic complexity of wheat and explaining the challenges associated with its systematic exploitation.

**Supplementary Information:**

The online version contains supplementary material available at 10.1007/s00122-026-05247-0.

## Introduction

The systematic exploitation of heterosis, the superior performance of hybrids compared to their parents, is a key strategy for improving crops such as maize, sugar beets, rice, rapeseed, and rye (Paterniani 2001; Zhoulin Gu and Han Bin 2024; Paril et al. 2024; Hochholdinger and Yu 2025; Revell et al. 2025). Various attempts have been made over the past decades to apply hybrid breeding to wheat, but these efforts have only met with limited success (Gupta et al. 2019; Revell et al. 2025; Abdullah et al. 2025). The main challenges stem from the high costs associated with producing hybrid seeds, which are driven by wheat's self-pollinating nature and must be offset by a sufficient level of heterosis (El Hanafi et al. 2022; Pathak et al. 2024; Schmidt et al. 2025; Revell et al. 2025).

To efficiently utilize heterosis, it is important to have a thorough understanding of its genetic basis. Three key genetic hypotheses have been proposed to explain heterosis (Lippman and Zamir 2007; Chen 2013; Hochholdinger and Baldauf 2018). The dominance hypothesis suggests that heterosis results from the complementation of deleterious recessive alleles at multiple loci (Bruce 1910; Keeble and Pellew 1910; Jones 1917; Collins 1921). In contrast, the overdominance hypothesis posits that heterozygosity at individual loci results in superior performance compared to the homozygous states (Crow 1948; Houle 1994). A related phenomenon is pseudo-overdominance, which occurs due to tightly linked loci with beneficial or superior dominant alleles in repulsion phase (Crow 2000). The epistasis hypothesis argues that favorable interactions between loci, rather than dominance at multiple loci, are the primary drivers of heterosis (Richey 1942; Schnell and Cockerham 1992). Overall, evidence from the literature indicates that these hypotheses are not mutually exclusive (Hua et al. 2003; Lippman and Zamir 2007).

A quantitative genetic framework was developed and implemented to study heterosis of approximately 1600 wheat single-cross hybrids generated by crossing 135 diverse elite lines adapted to Central Europe (Jiang et al. 2017). The analysis revealed that epistasis primarily drives heterosis in wheat. A subsequent study, conducted on a population of 1655 single-cross hybrids, generated by crossing 217 diverse elite lines adapted to Central Europe, confirmed these findings (Boeven et al. 2020). Interestingly, however, dominance effects became more pronounced in 152 wide crosses between elite lines and wheat genetic resources (Boeven et al. 2020). A computationally enhanced version of the quantitative genetic framework enabled a more thorough examination of the genetic basis of heterosis with a larger population comprising 5234 hybrids and 597 parental lines adapted to Central Europe (Li et al. 2025). This expanded analysis uncovered several heterotic QTL regions that act as epistatic hubs.

Mapping heterotic quantitative trait loci (QTL) in hybrids derived from multiple diverse parental lines can enhance mapping resolution by increasing allelic diversity and recombination. This advantage depends on sufficient population size or additional rounds of intercrossing. It may also be accompanied by low minor allele frequencies, which can limit the detection of heterotic QTL (Jiang et al. 2017). In contrast, biparental populations maximize dominance variance and provide high power to study the genetic basis of heterosis (Huo et al. 2023). Nevertheless, mapping resolution is often constrained in a biparental population derived from a single meiosis, such as F_1_-derived doubled haploids. Although alternative designs, such as advanced intercross populations, can increase recombination and improve mapping resolution (Darvasi and Soller 1995), this varies among biparental population types. Multi-parental populations, including nested association mapping (NAM) (Yu et al. 2008) and multi-parent advanced generation intercross (MAGIC) populations (Huang et al. 2012; Mackay et al. 2014; Scott et al. 2021), represent powerful alternatives that can balance mapping resolution and QTL detection power. Notably, NAM populations have already been implemented in wheat and successfully used for QTL mapping (Wingen et al. 2017; Wang et al. 2019; Sallam et al. 2020; Hu et al. 2022; Wright et al. 2024).

In addition to or as a complement to enhancing heterosis, reducing the costs associated with hybrid seed production is also a key target (Whitford et al. 2013; Gupta et al. 2019; Revell et al. 2025). The three main hybrid seed production systems currently in use or under exploration—chemical male sterility (CHA Croisor® 100, ASUR Plant Breeding SAS), cytoplasmic male sterility (CMS) (Farooq et al. 2023), and genic male sterility approaches such as the BLA (blue-aleurone) system (Darvey et al. 2019)—all rely on a reliable pollen supply. This challenge is particularly pronounced in CMS systems, which require a consistent pollen supply not only for hybrid production, but also for maintaining female CMS lines. Ideal male lines exhibit high anther extrusion, release abundant and viable pollen, and are taller than their female counterparts (Pickett 1993; Langer et al. 2014; Boeven et al. 2018; Garst et al. 2023). A recent study by Zapata et al. (2025) emphasized the complex interactions among individual trait components that influence the pollination capability of a genotype. Therefore, reliable indirect traits could not be suggested, leaving measurements of hybrid seed set from hybrid production trials as the standard.

This study is based on 10 multi-biparental population derived by crossing nine Central European elite lines, all derived from parents known for their pronounced anther extrusion. These populations were evaluated in two series, Series I and Series II. The objectives were to (i) identify male floral and agronomic traits, including visual anther extrusion (VAEX), plant height, heading date, and flowering time, that can predict seed set in wheat hybrids; (ii) evaluate hybrid performance in terms of grain yield, plant height, heading date, and yellow and leaf rust severity; and (iii) quantify heterosis for these traits and investigate its genetic basis through the joint analysis of dominance and epistatic effects.

## Material and methods

### Plant materials, genotyping, and genotypic data analyses

This study is based on 294 doubled haploid (DH) lines derived from 10 biparental populations, which were generated through non-orthogonal crosses among nine Central European elite winter wheat varieties (Table S1). Several parental lines are shared across multiple biparental populations, resulting in a set of interconnected families. The DHs were produced in two batches, referred to as Series I and Series II. The sizes of the biparental populations in Series I ranged from 7 to 61, while in Series II, they varied from 21 to 33. DH lines derived from the same biparental population were kept within the same series, and no populations were split across Series I and Series II. This structure also creates genetic connectivity between the two series. In addition to these, six checks were included to facilitate comparative analysis.

F_1_ hybrids were generated by crossing each DH line with one of its corresponding parental lines used as female testers. As a result, each hybrid shares one parental genome with the DH line and is expected to be heterozygous at approximately 50% of the loci segregating between the original parents.

Genomic DNA was extracted from two weeks-old single plants of the DHs and their respective parental lines, following the method described by Poursarebani et al. (2020). Genotyping was performed using the optimized wheat 25 K Infinium iSelect SNP array (SGS Institute Fresenius GmbH TraitGenetics Section, Gatersleben, Germany), which includes 24,145 single nucleotide polymorphisms (SNPs) distributed across the wheat genome. Marker positions were aligned to the Chinese Spring reference genome (Zhu et al. 2021). SNPs with a missing rate less than 90% were retained and missing values were subsequently imputed using Beagle (v5.1). After imputation, SNPs with a minor allele frequency (MAF) ≥ 0.05 were selected. Additionally, markers exhibiting observed heterozygosity greater than 0.05 were excluded, resulting in a total of 14,634 high-quality SNPs used for downstream analyses. A principal component analysis (PCA) was conducted using genotypic data from the nine parental lines. Subsequently, the DHs were projected onto the same principal components by centering their genotypes using the parental means and applying the loadings (rotation matrix) obtained from the parental PCA (Abdi and Williams 2010).

### Hybrid seed set trials and phenotyping

The two series of DH lines were evaluated alongside their parental lines to assess pollination capability in field trials conducted at Verneuil l'Étang, Île-de-France, France (latitude 48° 35′09″N, longitude 2°52′18″E) during the 2021/2022 (Series I) and 2022/2023 (Series II) growing seasons. To facilitate hybridization, the lines were arranged in crossing blocks following an unreplicated alpha lattice design (Patterson and Williams 1976). Each crossing block was isolated by an early-flowering triticale line to prevent pollen flow between neighboring blocks. The crossing blocks were randomized into 15 incomplete blocks, each containing 10 DH lines. Within each crossing block, the two parents of the respective DH population were used as female plants, while the DH line was positioned on each side as male plants to ensure maximum pollen supply (Supplementary Figure S1). Plot sizes were 6.0 m^2^. Sowing density was 200 grains m^−2^ for male lines and 400 grains m^−2^ for female lines. The female lines were emasculated using a chemical hybridization agent (CHA) provided by a commercial breeding program. The CHA was applied when the flag leaf ligule became just visible. Sterility was assessed by bagging up to 10 spikes per plot immediately after CHA application. To ensure representative sampling, the bags were placed at the beginning, middle, and end of each plot. The efficacy of the CHA treatment on the female lines was assessed by calculating sterility values, which entails counting the number of kernels per bag and dividing it by the number of sampled ears, following the methodology described in Zapata et al. (2025). In this study, a fixed threshold of 4 kernels per bag was applied to identify and exclude plots with male-fertile lines, ensuring accurate assessment of male sterility. Although not directly assessed, the potential for cross-pollination between adjacent female plots due to incomplete sterility was considered to be low, as female plots were flanked by taller and highly pollen-producing DH lines.

The following traits were assessed in the hybrid seed production trials. Although measured in both female and male parental lines, the data presented here correspond to the male parental lines. Plant height (PH) was determined in cm by measuring from the ground to the tip of the spikes, excluding awns, following the protocol described by Langer et al. (2014). Heading date (HD) was defined as the number of days elapsed since January 1 st until 75% of all ears had fully emerged from the flag leaf (Zadok stage 59). Flowering begin **(**FT_begin_**)** and flowering end (FT_end_) were both expressed in days after January 1st. In male parental lines, FT_begin_ was recorded as the date when the first anther extruded (Zadok stage 61), and FT_end_ as the date when yellow anthers were no longer visible (Zadok stage 69). In contrast, for female lines, flowering start was visually assessed at the onset of floret opening, and flowering end was considered when florets had fully closed again, following Schmidt et al. (2025). Flowering duration (FT_dur_) was then derived as the number of days between FT_begin_ and FT_end_. In addition to these traits, the following measurements were recorded exclusively for male parental lines: Visual anther extrusion (VAEX) was monitored during the entire flowering period using a modified version of the 1–9 scale proposed by Langer et al. (2014), with the adapted scale ranging from 0 (no visible anthers) to 9 (maximum extrusion). Given that white anthers are no longer capable of releasing viable pollen (Impe et al. 2020), only yellow anthers were scored. Based on these observations, the maximum (VAEX_max_), average (VAEX_mean_), and total accumulated (VAEX_sum_) VAEX values were derived. Hybrid seed set (Seed Set, g m⁻^2^) was quantified as the grain weight harvested from each cross-pollinated hybrid seed plot, expressed in g m^−2^ at 14% moisture content.

After sterility curation, 18 plots (6% of Series I) and 70 plots (23% of Series II) were removed. An additional filtering step was then applied to ensure flowering synchrony between male and female parents. As a result, 20% of the plots in Series I were further excluded, whereas no additional plots were removed from Series II, which exhibited good flowering synchrony. Subsequently, best linear unbiased predictions (BLUPs) for seed set were estimated from the curated dataset by fitting the following linear mixed model:

1 $$Y_{ij} = \mu { } + { }F_{i} + { }M_{j} + { }\varepsilon_{ij} ,$$

where ${Y}_{ij}$ represents the seed set observed for the combination of the *i*-th female and *j*-th male line, $\mu$ is the overall mean, ${F}_{i}$ denotes the effect of the female line, ${M}_{j}$ the effect of the male line, and ${\varepsilon }_{ij}$ the residual error term. The female line was treated as a fixed effect, while the male line was considered random effect. This model structure enabled the evaluation of male performance through their contribution to seed set in plots of female lines, and the resulting BLUPs were used in subsequent analyses.

For traits recorded in male lines (PH, HD, FT_begin_, FT_end_, VAEX), statistical analyses were performed using averages across plots of each male line. These values were used to represent the performance of the male parental lines in subsequent analyses.

To identify potential indirect predictors of seed set, pairwise partial correlations (R) were calculated between seed set and male parental traits separately for Series I and Series II. For each trait pair, a baseline model (Seedset ~ Pedigree) was compared with an extended model (Seedset ~ Pedigree + Traitⱼ) using F-tests. The partial correlation was derived from changes in model fit and reflects the relationship between traits after accounting for pedigree effects. The pedigree refers to the parental cross used to generate the DH lines (e.g., KWS Livius × Bussard). Statistical significance was assessed via F-tests, with p-values adjusted for multiple testing using the Benjamini–Hochberg false discovery rate (FDR) (Benjamini and Hochberg 1995) correction within each series. All analyses were performed in R (v4.2.2).

### Genomic heritability-based assessment of genomic–phenotypic consistency

To ensure data accuracy and control for potential inconsistencies arising from mislabeling, seed mixtures, or DNA contamination, the concordance between genomic and phenotypic data was evaluated through genomic heritability estimates, using plot means for male parental traits and BLUPs for Seed Set. Five-fold cross-validation was employed, randomly splitting the data into training and validation sets, and this process was repeated 100 times. In each cross-validation run, the phenotypes of the validation set were masked, and the following model was fitted to obtain the genomic best linear unbiased predictions (GBLUP):

2 $$y = X\beta + g_{A} + e,$$

where $y$ denotes the *n*-dimensional vector of the design-corrected phenotypic data from a particular environment, $\beta$ is the *k*-dimensional vector of fixed effects, $X$ is the corresponding $n\times k$ design matrix, which in this case correspond only to the common population mean. ${g}_{A}\sim N(0,{G}_{A}{\sigma }_{g}^{2})$ is the *n*-dimensional random vector of (additive) genetic values, where ${G}_{A}$ is the corresponding genomic relationship matrix derived from marker information, and ${\sigma }_{g}^{2}$ is the corresponding genetic variance component. $e\sim N(0,I{\sigma }_{e}^{2})$ corresponds to the vector of residuals, $I$ is the identity matrix, and ${\sigma }_{e}^{2}$ is the residual variance component. Here, $G_{A} = ZZ^{\prime}$ is the additive genomic relationship “VanRaden” G-matrix (VanRaden 2008), where $Z=M/\sqrt{c}$, $M$ is the $n\times s$ matrix of marker profiles coded as $2-2p$, $1-2p$ and $-2p$ ($p$ is the allele frequency), $c={\sum }_{i=1}^{s}2{p}_{i}(1-{p}_{i})$, and $s$ is the number of markers. In each cross-validation run, the genomic prediction ability was defined as the correlation between observed phenotypes and those predicted by the Eq. 2 for the genotypes in the validation set. GBLUP was implemented using the BWGS package (Charmet et al. 2020) in R environment (version 4.2.2).

### Genome-wide association mapping in the hybrid seed set trials

To dissect the genetic basis of seed set, genome-wide association mapping (GWAS) was performed separately in Series I and Series II. After filtering for sterility and flowering asynchrony, a total of 135 and 133 DH lines were retained for GWAS analyses in Series I and Series II, respectively. The analyses were based on 14,634 high-quality SNPs. GWAS was performed using the GWAS() function from the “rrBLUP” R package (Endelman 2011), incorporating the first five principal components (PCs, n.PC = 5) as fixed covariates. To identify significant associations between markers and traits, a multiple-testing-corrected significance threshold was calculated using the Bonferroni–Holm method (Holm 1979). The threshold was defined as 0.05 divided by the effective number of independent SNPs, which was estimated based on the number of principal components required to explain at least 99.5% of the cumulative genetic variance, following the method proposed by Gao et al. (2008).

### Evaluating hybrid performance in field trials

The hybrids resulting from the hybrid seed set trials, i.e., DHs × Parent-1 and DHs × Parent-2, as well as the DHs and their parents were evaluated in field trials during the 2022/2023 and 2023/2024 growing seasons in Series I, and during 2023/2024 and 2024/2025 in Series II. High-quality hybrid seed from other sources was used for some hybrid combinations for which production failed. Trials were conducted using an alpha lattice design with two replications. Genotypes from Series I were evaluated across up to nine environments (combination of location, year, and plot type (such as, grain yield plot or observation plot)): three in Gatersleben, Germany (51.826° N, 11.281° E); two in Schackstedt, Germany (51.716° N, 11.616° E); two in Biendorf, Germany (51.737° N, 11.844° E); and two in Jerxheim, Germany (52.084° N, 10.900° E). Genotypes from Series II were tested in up to six environments: two in Gatersleben, Germany (51.826° N, 11.281° E); two in Cochstedt, Germany (51.874° N, 11.388° E); and two in Biendorf, Germany (51.737° N, 11.844° E) (Supplementary Table S8). Plot sizes for the yield trials ranged from 7.5 to 8.7 m^2^. Plant height (PH) and heading date (HD) were recorded following the same protocols as used in hybrid seed set trials. Grain yield (GY, Mg ha^−1^) was calculated from the grain weight harvested per plot and adjusted to a standard moisture content of 14%. Additionally, yellow rust (YR) and leaf rust (LR) severity were assessed in plots of 0.50 m^2^ using a standardized 1–9 scale (Bundessortenamt 2000), where higher values indicate greater disease severity and lower values indicate reduced severity.

Prior to statistical analyses, data cleaning and quality control were conducted in two stages: within environments and across environments. Within each environment, the following linear mixed model was fitted:

3 $$Y_{ijkl} = \mu + G_{i} + g_{j\left( i \right)} + r_{k} + b_{l\left( k \right)} + \varepsilon_{ijkl} ,$$

where ${Y}_{ijkl}$ is the phenotypic value of genotype $j$ within group $i$ in replication $k$ and block $l$; $\mu$ is the overall mean; ${G}_{i}$ is the fixed effect of the group (such as hybrid, line, or check); while genotypes within groups ${(g}_{j(i)})$, replications ${r}_{k}$, and blocks nested in replication ${b}_{l\left(k\right)}$ were treated as random effect.

For outlier identification, residuals were first standardized using the rescaled median absolute deviation (MAD) and then converted to two-sided *p*-values under the standard normal distribution. *P*-values were adjusted for multiple testing using the Holm–Bonferroni procedure (*α* = 0.05), following the BH-MADR approach (Bernal-Vasquez et al. 2016). Outliers were identified and subsequently removed, and the model refitted using the cleaned dataset to assess data quality and obtain best linear unbiased estimates (BLUEs) within each environment. The repeatability (${R}_{n}$) within environments was estimated for hybrids, checks, and parental lines as follows:

4 $$R_{n} = \frac{{\sigma_{{\mathrm{g}}}^{2} }}{{\sigma_{{\mathrm{g}}}^{2} + \frac{{ \sigma_{e}^{2} }}{{n_{{\mathrm{r}}} }} }},$$

where ${\sigma }_{\mathrm{g}}^{2}$ and ${\sigma }_{e}^{2}$ represent the genotypic and residual variances, and ${n}_{\mathrm{r}}$ is the number of replications.

Across environments, variance components were estimated by fitting the following linear mixed model:

5 $$Y_{ijklm} = \mu + G_{i} + g_{j\left( i \right)} + e_{k} + \left( {ge} \right)_{j\left( i \right)k} + r_{l\left( k \right)} + b_{{m\left( {kl} \right)}} + \varepsilon_{ijlkm} ,$$

where ${Y}_{ijklm}$ is the observed phenotypic value of the *j*-th genotype within group $i$ (hybrid, line, or check) in the *k*-th environment, within the *l*-th replication and the *m*-th block. $\mu$ is the overall mean; ${G}_{i}$ represents the group effect; ${g}_{j(i)}$ the genotypic effect nested within group; ${e}_{k}$ the environmental effect; ${(ge)}_{j(i)k}$ the genotype-by-environment interaction; ${r}_{l\left(k\right)}$ and ${b}_{m\left(kl\right)}$ the replication and block effects, respectively; and ${\varepsilon }_{ijlkm}$ the residual error. The group effect was fitted as fixed, while all other effects were treated as random. Outlier detection across environments followed the same procedure described above for the within-environment analysis. After removing outliers, the model (Eq. 5) was refitted to obtain BLUEs, fitting genotype as a fixed effect and omitting the group term, while all other effects were assumed as random. For each trait, the broad-sense heritability (*H*) was derived from variance components as follows:

6 $$H = \frac{{\sigma_{{\mathrm{g}}}^{2} }}{{\sigma_{{\mathrm{g}}}^{2} + \frac{{\sigma_{{{\mathrm{g}} \times {\mathrm{e}}}}^{2} }}{{n_{{\mathrm{e}}} }} + \frac{{\sigma_{{{\mathrm{error}}}}^{2} }}{{n_{{\mathrm{e}}} \times n_{{\mathrm{r}}} }}}},$$

where ${\sigma }_{\mathrm{g}}^{2}$ denotes to the genetic variance due to genotypes, ${\sigma }_{e}^{2}$ represents the environment variance, ${\sigma }_{\mathrm{g}\times \mathrm{e}}^{2}$ represents the variance of genotype-by-environment interaction, ${n}_{\mathrm{e}}$ is the number of environments, $\sigma^{2}_{{{\mathrm{error}}}}$ corresponds to the residual variance, and ${n}_{\mathrm{r}}$ is the number of replications.

Within each series, to estimate the heritability of mid-parent heterosis (MPH) for grain yield, plant height, heading date, yellow rust, and leaf rust, MPH values were first calculated on the basis of the BLUEs of hybrids and their parents within each environment. Additionally, better-parent heterosis (BPH) was calculated as the difference between the F_1_ and its best-performing parent. A linear mixed model was then fitted to the MPH values, including random genotype and environment effects, without genotype-by-environment interaction effects since confounded with the residuals. Finally, the heritability of MPH was estimated by using the formula as:

7 $$H_{{{\mathrm{mph}}}} = \frac{{\sigma_{{{\mathrm{gmph}}}}^{2} }}{{\sigma_{{{\mathrm{gmph}}}}^{2} + \frac{{\sigma_{{{\mathrm{error}}}}^{2} }}{{n_{{\mathrm{e}}} }}}}{ },$$

where the notations are same as in (6) except that ${\sigma }_{gmph}^{2}$ represents the MPH variance due to genotypes.

All linear mixed models for data curation and phenotypic analyses were implemented in the ASReml-R package (v4.2; Butler et al. 2023).

### Genome-wide association mapping for heterotic effects and dominance effects

The DH lines and parental lines were directly genotyped using a SNP array as mentioned before. For the hybrids derived from crosses between DH lines and parental lines, genotypes were inferred from the parental genotypic data, which is a commonly used approach when working with homozygous parental lines. After retaining as much high-quality data as possible, a total of 296 hybrid combinations in Series I and 292 hybrid combinations in Series II were retained for GWAS analysis of genetic basis of heterosis. To assess population structure, principal component analysis (PCA) was performed based on the genotypic data of the F_1_ hybrids in Series I and Series II, separately.

**Fig. 1:**
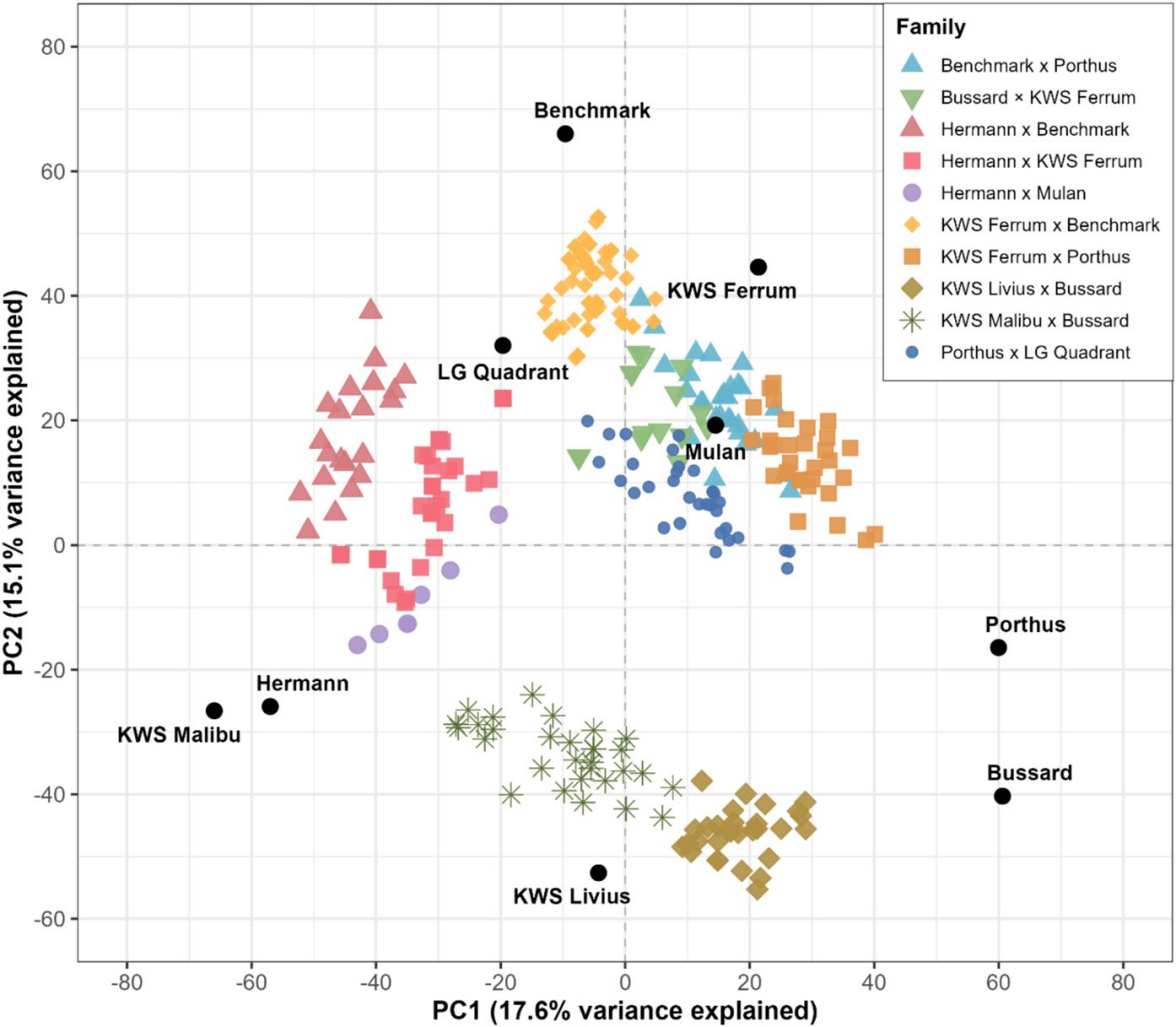
Principal component analysis (PCA) of the combined population including nine parental lines and their 294 doubled haploid (DH) progeny

Following the definition of the heterotic effect of a marker as its net contribution to MPH, both its dominance effect and its digenic epistatic interactions with the entire genetic background should be considered (Jiang et al. 2017). To this end, a recent one-dimensional genome-wide scanning approach, hQTL-ODS (Li et al. 2025) model, was applied to detect heterotic quantitative trait loci (hQTL) within each series. Briefly, for each marker, the following two linear mixed models are compared.

8 $$\boldsymbol{y}_{{MPH}} = \boldsymbol{X}\alpha + \boldsymbol{g}_{D} + \boldsymbol{g}_{{AA}} + \boldsymbol{g}_{{AD}} + \boldsymbol{g}_{{DD}} + \boldsymbol{\varepsilon },$$

9 $${\boldsymbol{y}}_{MPH} = {\boldsymbol{h}}_{i} + \boldsymbol{X\alpha } + {\boldsymbol{g}}_{D} + {\boldsymbol{g}}_{{{\mathrm{AA}}}} + {\boldsymbol{g}}_{{{\mathrm{AD}}}} + {\boldsymbol{g}}_{{{\mathrm{DD}}}} + {\boldsymbol{\varepsilon}},$$

where ${{\boldsymbol{y}}}_{\mathrm{MPH}}$ is the vector of mid-parent heterosis (MPH) for all hybrids and ${\boldsymbol{\varepsilon}}$ is the residual. Model (8) is the null model containing covariate effects (${\boldsymbol{X}}\boldsymbol{\alpha }$), in our study where ${\boldsymbol{X}}$ is the design matrix for DH family information and $\boldsymbol{\alpha }$ is the corresponding effects (Fig. 1 and Table 1), and multiple genetic background effects ${{\boldsymbol{g}}}_{D}\sim N(0,{{\boldsymbol{K}}}_{D}{\sigma }_{D}^{2})$, ${{\boldsymbol{g}}}_{\mathrm{AA}}\sim N(0,{{\boldsymbol{K}}}_{\mathrm{AA}}{\sigma }_{\mathrm{AA}}^{2})$, ${{\boldsymbol{g}}}_{\mathrm{AD}}\sim N(0,{{\boldsymbol{K}}}_{\mathrm{AD}}{\sigma }_{\mathrm{AD}}^{2})$, and ${{\boldsymbol{g}}}_{\mathrm{DD}}\sim N(0,{{\boldsymbol{K}}}_{\mathrm{DD}}{\sigma }_{\mathrm{DD}}^{2})$, where ${{\boldsymbol{K}}}_{*}$ ($*$ denotes D, AA, AD, or DD) is the genomic kinship matrix that accounts for a specific type of genetic background effects (depending on $*$) and ${\sigma }_{*}^{2}$ is the corresponding variance component. The alternative model (9) includes the heterotic effect (${{\boldsymbol{h}}}_{i}$) of the tested marker in addition to all effects in the null model. The heterotic effect ${{\boldsymbol{h}}}_{i}$ is defined as a complex linear combination of (4 $p$−3) effects, i.e., the dominance effect ${d}_{i}$ and the epistatic effects ${aa}_{ij}$(additive-by-additive), ${ad}_{ij}$(additive-by-dominance), ${da}_{ij}$(dominance-by-additive), ${dd}_{ij}$ (dominance-by-dominance, $j=1,\dots ,p$ and $j\ne i$, where $p$ is the number of markers) (Jiang et al. 2017). And ${{\boldsymbol{h}}}_{i}$ is treated as random effect following a multi-variate normal distribution, ${{\boldsymbol{h}}}_{i}\sim N(0,{{\boldsymbol{H}}}_{i}{\sigma }_{i}^{2})$, where covariance matrix ${{\boldsymbol{H}}}_{i}$ is constructed based on the definition of ${{\boldsymbol{h}}}_{i}$ and ${\sigma }_{i}^{2}$ is the corresponding variance component. The likelihood ratio test is used to assess the significance of ${{\boldsymbol{h}}}_{{\boldsymbol{i}}}$.

**Table 1:** Summary of seed set and indirect traits across up to 300 doubled haploid (DH) lines (Series I and II) for seed set and male traits. The genomic heritability (h^2^_Gen_) and the pairwise partial correlations (R) between the seed set and the other traits are also presented for Series I and Series II. The following traits were recorded: plant height (PH, cm), heading date (HD, days after January 1 st), flowering begin (FT_begin_, days), flowering end (FT_end_, days), flowering duration (FT_dur_, days), visual anther extrusion scores: total (VAEX_sum_), mean (VAEX_mean_), and maximum (VAEX_max_), and hybrid seed set of the DHs when crossed with their parents (Seed set, g m^−2^)

Trait	Seed set	PH	HD	FT_begin_	FT_end_	FT_dur_	VAEX_sum_	VAEX_mean_	VAEX_max_
*Series I*									
Mean	169.8	96.8	137.9	136.7	146.6	9.9	14.4	1.0	3.5
*h*^2^_Gen_	0.38	0.54	0.56	0.65	0.59	0.33	0.41	0.43	−0.02
*R*	–	0.32***	0.11	0.02	0.14	0.16	0.23**	0.23**	0.03
*Series II*									
Mean	423.9	133.0	140.7	145.7	152.2	6.6	7.7	1.5	3.2
*h*^2^_Gen_	0.20	0.47	0.31	0.47	0.42	0.17	0.46	0.36	0.47
*R*	–	−0.01	0.35***	0.31***	0.38***	−0.03	0.05	0.04	0.07

^*^,^**^,^***^ refers to *P* ≤ 0.05, 0.01, 0.001, respectively

To further clarify the contribution of the dominance effect to heterosis, we conducted a significance test of the dominance effect using a linear mixed model as following.

10 $${\boldsymbol{y}}_{MPH} = \boldsymbol{X\alpha } + {\boldsymbol{m}}_{{\boldsymbol{i}}} d_{i} + {\boldsymbol{g}}_{D} + {\boldsymbol{\varepsilon}},$$

where all notation same as in (8) except that ${d}_{i}$ denotes the dominance effect of the *i*-th marker, and ${{\boldsymbol{m}}}_{{\boldsymbol{i}}}$ is the linearly transformed design matrix for the dominance effect (Li et al. 2025).

In both heterotic effect and dominance effect, the significance threshold was determined to be *p*-value < 0.05 after correction based on 500 permutation tests (Churchill and Doerge 1994).

For candidate gene identification, genomic regions spanning ± 1 Mb around each significant SNP were extracted based on their physical positions in the *Triticum aestivum* reference genome (IWGSC RefSeq v2.1). All genes located within these intervals were evaluated through literature review to identify potential associations with yield-related processes.

## Results

### Genetic structure of DH lines reflects parental diversity

A principal component analysis was performed based on 14,634 high-quality SNPs to assess the genetic structure of the 294 DH lines derived from crosses among nine Central European elite lines (Fig. 1). The nine lines covered a broad diversity space and are widely distributed along the first two principal components. As we expected, the analysis revealed that the DH lines from the same family clustered together and are placed between their corresponding parental lines.

### Divergent profiles of putative indirect traits for seed set between the two series

The seed set in the F_1_ seed production trial exhibited a broad phenotypic range and moderate genomic heritability (0.38 in Series I and 0.20 in Series II; Table 1). Wide phenotypic variation was also observed for putative indirect traits at the F_1_ seed production trial, yielding moderate to high genomic heritability (0.33–0.65 in Series I and 0.31–0.47 in Series II). The exceptions were VAEX_max_ in Series I (−0.02) and FT_dur_ in Series II (0.17), which showed lower estimates. Given assessing seed set is costly, we explored indirect predictors and identified pronounced differences in partial correlation patterns between the two series. In Series I, plant height, VAEX_sum_, and VAEX_mean_ showed significant predictive value. Notably, the height differential between parents was also a strong predictor (*r* = 0.31, *P* < 0.001), highlighting the importance of relative plant height for pollination success. In Series II, HD, FT_begin_, and FT_end_ emerged as key correlated traits. These findings suggest that indirect selection strategies for seed set should be tailored to the population in question, reflecting the different genetic structures that underlie reproductive performance. However, as the two series were evaluated in different growing seasons and involve distinct sets of DH lines, these differences may also reflect environmental variation or genotype-by-environment interactions. Therefore, the observed trait associations should be interpreted as a combination of population-specific genetic effects and environmental influences.

### Seed set QTL differ between the two series

**Fig. 2:**
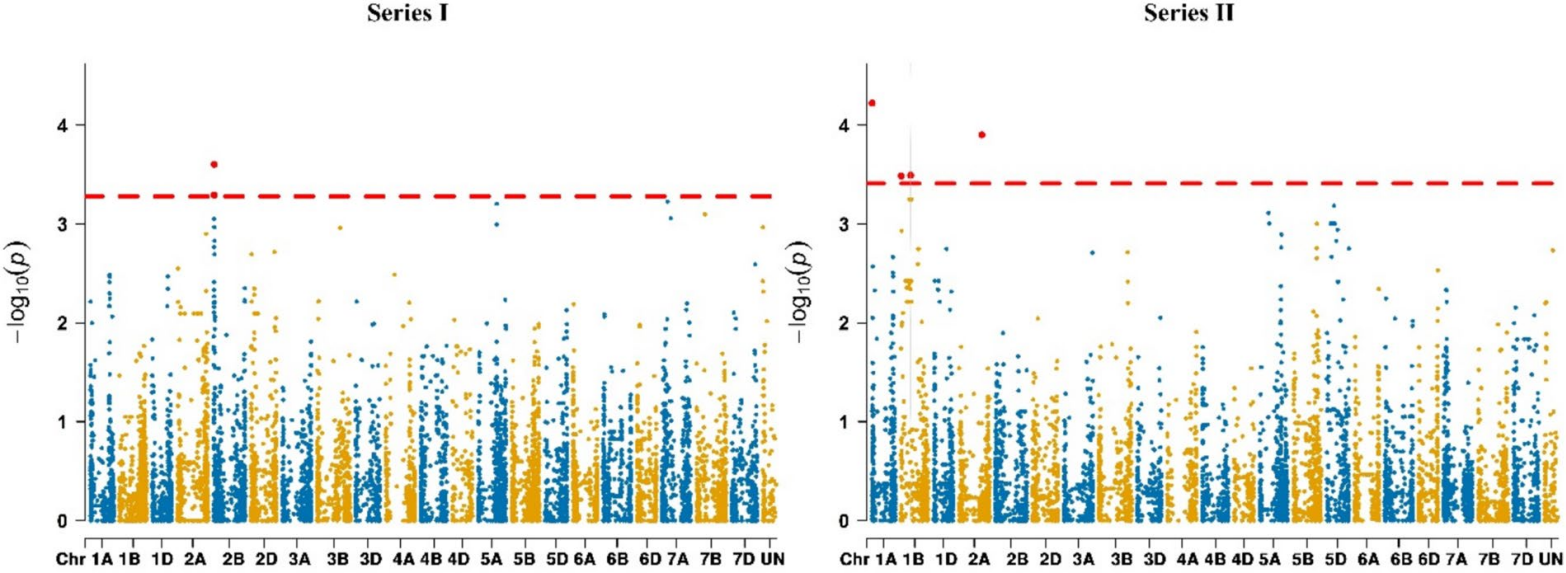
Manhattan plots from association mapping scans in Series I and Series II evaluated for their seed set (g m⁻^2^). The y-axis represents − log₁₀(*p*), while the x-axis shows the physical positions (Mb) of high-quality SNPs along the chromosome sequence maps of wheat RefSeq v2.1 (Chinese Spring; Zhu et al. 2021). Significant marker–trait associations above the significance threshold are indicated

To dissect the genetic architecture of seed set, a genome-wide association study was conducted in the F_1_ seed set trials. A total of 479 significant marker–trait associations (MTA) were identified, with 180 in Series I and 299 in Series II, involving both seed set and indirect traits (Supplementary Table S3). Notably, four markers on chromosome 2B were significantly associated with seed set in Series I (Fig. 2), collectively explaining 5% of the phenotypic variation. In Series II, additional significant associations were detected on chromosomes 1 A, 1B (two MTA), and 2 A, explaining 18.5% of the variation in total. No overlapping markers were detected between Series I and Series II, suggesting distinct genetic architectures underlying seed set in the two populations.

A total of 471 significant marker–trait associations were detected for the putative indirect traits, with 176 in Series I and 295 in Series II. These associations explained between 7% of the phenotypic variation for FT_dur_ (Series I) and up to 67% for plant height (PH) (Series II). However, no overlapping markers were detected between seed set and indirect traits, suggesting distinct genetic architectures underlying direct seed set and its correlated traits. Considering a ± 1 Mb window around each seed set QTL, no significant associations with indirect traits were detected in close genomic proximity. In Series I, the closest indirect-trait association (AX-95160129 at 648.811 Mb), corresponding to VAEX_mean_ and VAEX_sum_, was located approximately 633–638 Mb away from the seed set lead SNPs (10.886–15.741 Mb), with negligible linkage disequilibrium (*r*^2^ = 0.061–0.066). In Series II, the nearest pair, tplb0025b13_2687 associated with seed set and located at 4.38 Mb, and CAP12_c3074_192 associated with PH and located at 5.05 Mb on chromosome 1 A, was within 1 Mb but exhibited extremely low linkage disequilibrium (*r*^2^ = 0.004).

### Beneficial mid-parent heterosis and moderate to high heritability

The hybrids generated in the production trial were evaluated together with their parents in multi-environmental field trials. The genetic variance (${\sigma }_{\mathrm{g}}^{2}$) was significantly (*p* < 0.001) larger than zero for grain yield, heading date, and plant height in both series, as well as for yellow rust in Series I and leaf rust in Series II (Supplementary Table S2). Heritability ranged from moderate to high (*H*^2^ = 0.52–0.97). In Series I, grain yield (GY) and yellow rust (YR) exhibited moderate heritability (*H*^2^ = 0.52–0.54), whereas heading date (HD) and plant height (PH) showed high heritability (*H*^2^ = 0.90–0.96). In contrast, GY in Series II had higher heritability (*H*^2^ = 0.81 in lines, 0.87 in hybrids), comparable to HD and PH (*H*^2^ ≥ 0.88), while leaf rust (LR) exhibited slightly lower estimate (*H*^2^ = 0.68). The high heritability of hybrid performance, particularly for grain yield (GY), provides a reliable foundation for investigating mid-parent heterosis.

The absolute (relative) mid-parent heterosis (MPH) of grain yield varied widely from 0.0 (0.0%) to 1.8 Mg ha^−1^ (37%) in Series I, with a mean value of 0.7 Mg ha^−1^ (13.7%), and from −1.5 (−16%) to 1.6 Mg ha^−1^ (19%) in Series II, with a mean value of 0.5 Mg ha^−1^ (6.1%). In Series I, the highest MPH (1.8 Mg ha⁻^1^) was identified in the cross KWS Ferrum x 10DH_188, where a high phenotypic contrast between parents was observed (*P*1 = 5.7 vs. *P*2 = 3.9 Mg ha⁻^1^). Conversely, in Series II, top-performing crosses such as LG Quadrant x 10DHS2_90 and Porthus x 10DHS2_63 reached maximum absolute yields of 10.1 Mg ha⁻^1^. Notably, in Porthus x 10DHS2_63, parental phenotypic variance was zero (*P*1 = *P*2 = 8.8 Mg ha⁻^1^), showing that substantial heterotic gains (MPH = 1.4 Mg ha⁻^1^) were achieved even when both parental lines exhibited identical yield levels, surpassing the performance of these high-performing parents (Supplementary Table S9). Better-parent heterosis (BPH) for grain yield reached maximum values of 1.1 Mg ha⁻^1^ (19.1%) in Series I and 1.3 Mg ha⁻^1^ (17.2%) in Series II (Supplementary Table S9). The average MPH was lower for heading date (−0.5% in Series I and II) and plant height (8.4% and 0.9% in Series I and II). Consistent with this, BPH for plant height showed increases of up to 9.7 cm over the superior parent, while heading date remained stable with a maximum BPH of 2.9 days. For LR and YR maximum negative heterosis (indicating reduced susceptibility) reached −1.8 units (−35%) and −1.3 units (−23%), respectively (Table 2); notably, BPH for these traits reached negative values of up to −44.0% (Supplementary Table S9), highlighting the superior resistance of several hybrids compared to their most resistant parent. Heritability of mid-parent heterosis was high for heading date and plant height in both Series I and Series II, ranging from 0.75 to 0.92 (Table 2). Grain yield heterosis exhibited moderate heritability (0.53 in Series I, 0.63 in Series II), and yellow rust (YR) and leaf rust heterosis (LR) showed rather modest heritability (0.25 for YR in Series I and 0.5 for LR in Series II). Importantly, the relatively high heritability of MPH, together with the significant genetic variance (${\sigma }_{\mathrm{gmph}}^{2}$) for heterosis across all traits, provides a robust foundation for the genetic dissection of heterosis in these populations.

**Table 2:** Mean, minimum (min), and maximum (max) mid-parent heterosis (MPH) for each trait within two hybrid series (I and II), together with variance components and broad-sense heritability (*H*^2^) estimates for MPH. Traits: grain yield (GY, Mg ha^−1^), heading date (HD, days after January 1 st), plant height (PH, cm), and disease (yellow rust, YR, in Series I; leaf rust, LR, in Series II, 1–9 scale)

Trait	GY	HD	PH	YR
*Series I*				
Mean	0.7	−0.8	6.2	0.2
Min	0.0	−4.5	−1.0	−1.3
Max	1.8	1.7	16.2	1.2
${\sigma }_{\mathrm{gmph}}^{2}$	0.04 ***	1.05 ***	6.8 ***	0.05 **
*σ*^2^_E_	0.02 ***	0.11 ***	3.77 ***	0.07 ***
*σ*^2^_e_	0.07	1.18	11.09	0.61
*H*^2^	0.53	0.86	0.79	0.25
*Series II*				
Trait	GY	HD	PH	LR
Mean	0.5	−0.8	0.7	0.0
Min	−1.5	−7.0	−14.8	−1.8
Max	1.6	3.6	12.3	1.7
${\sigma }_{\mathrm{gmph}}^{2}$	0.13 ***	2.67 ***	16.76 ***	0.16 ***
*σ*^2^_E_	0.06 ***	0.23 ***	4.56 ***	0.07 ***
*σ*^2^_e_	0.22	1.07	27.62	0.64
*H*^2^	0.63	0.92	0.75	0.50

^*^,^**^,^***^ refers to P ≤ 0.05, 0.01, 0.001, respectively

### Genome-wide association mapping identifies loci underlying heterosis

The PCA results of F_1_ hybrids were fully consistent with the known pedigree relationships (as the legend in plot, Supplementary Figure S2 and Supplementary Figure S3), as expected, indicating that the genetic structure of the hybrids is well captured by the pedigree information. Therefore, both the pedigree-based covariates and the genotype-based population structure provide effective control for confounding in the genome-wide association study.

**Fig. 3:**
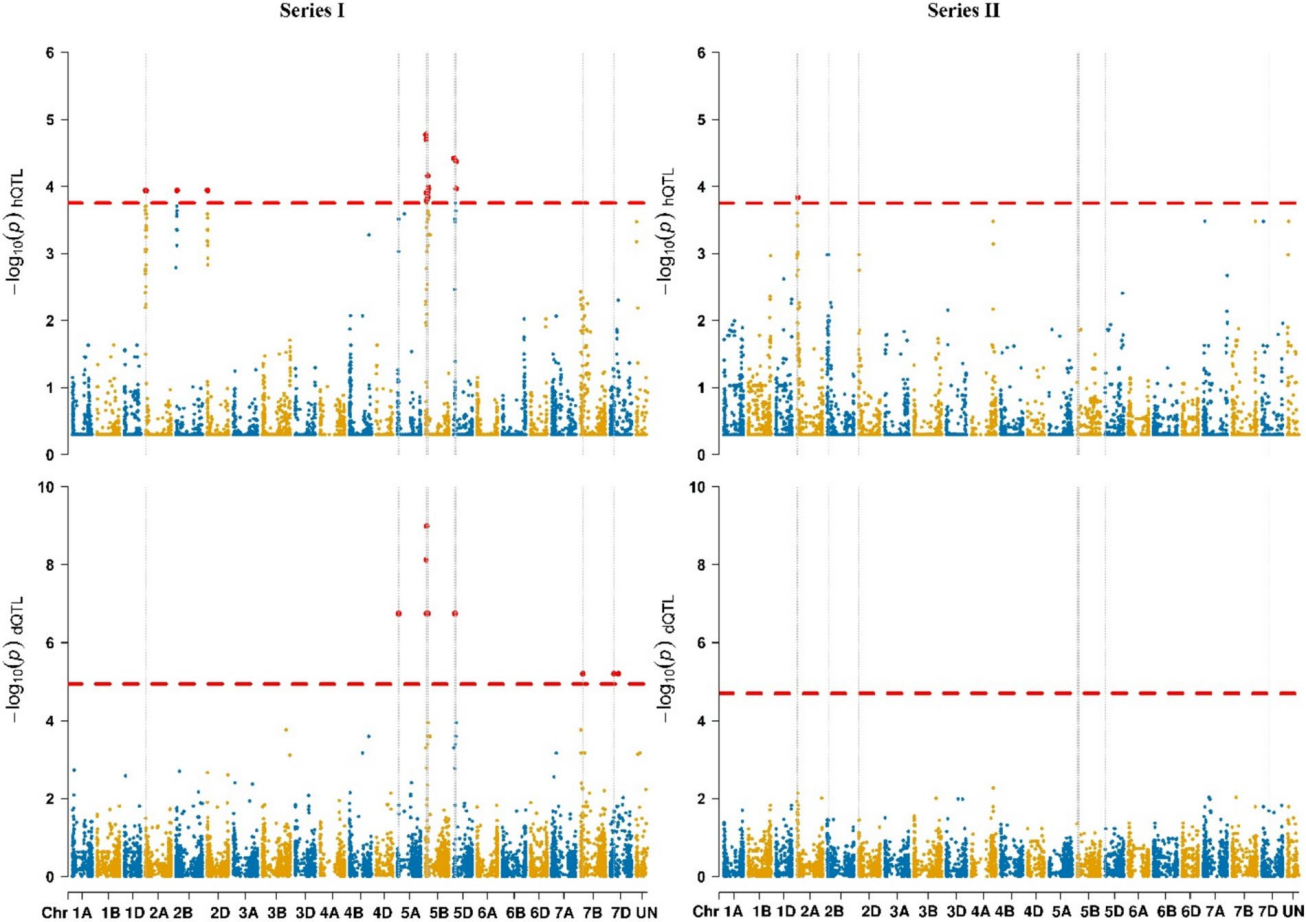
Manhattan plots of heterotic QTL (hQTL) and dominance QTL (dQTL) analyses for grain yield (GY) in Series I and Series II. The red dashed line indicates the significance threshold (*α* = 0.05) derived from 500 permutation tests. Significant marker–trait associations exceeding this threshold are highlighted. The scale of p-values corresponds to − log10(*p* -value), while positions (Mb) of 14,634 high-quality SNPs on the chromosome sequence maps are according to RefSeq 2.1 of Chinese Spring (Zhu et al. 2021)

Genome-wide association mapping was conducted to map heterotic QTL (hQTL) and their dominance components (dominance effects, dQTL). In Series I for grain yield, 17 significant MTA of heterotic effects were detected on chromosomes 2A, 2B, 2D, 5B, and 5D, and 28 MTA for dominance effects were detected on chromosomes 5A, 5B, 5D, 7B, and 7D (Fig. 3; Supplementary Table S4). Five MTAs overlapped between hQTL and dQTL, primarily on chromosome 5B. Of the five MTAs, two are located very close to each other near 21 Mb (wsnp_BF201102B_Ta_2_5 and BobWhite_rep_c50066_63), while the remaining three are located at approximately 27 Mb (AX-94830972), 54 Mb (IAAV3261), and 66 Mb (Tdurum_contig44130_228), respectively. The remaining 12 heterotic associations and 23 dominance associations were unique to each category, indicating that some loci contribute to heterosis primarily through both dominance and epistasis, while others are more likely by epistatic effects. In Series II, only one hQTL was identified on chromosome 2A, with no corresponding dominance association, suggesting that this hQTL is primarily driven by epistatic interaction effects. No overlapping associations were detected between the two series.

The analysis of genomic regions surrounding significant MTAs revealed a total of 754 candidate genes potentially associated with heterotic and dominance effects for grain yield. For heading date, two heterotic associations were detected in Series I on chromosomes 2A and 2D, with no corresponding dominance associations. In Series II, 77 heterotic associations and four dominance associations were identified (one on chromosome 7A and three on 7B). On chromosome 7B, three loci located around 1.0–1.3 Mb (GENE-2935_850, RAC875_c17182_600, and Kukri_rep_c71778_644) showed overlapping heterotic and dominance associations within < 1 Mb of each other. The remaining 74 heterotic associations did not overlap with dQTL, suggesting that epistatic interactions account for a large portion of heterotic variation for heading date heterosis (Supplementary Table S5; Supplementary Figure S4).

For plant height, nine heterotic associations were detected in Series I on chromosomes 4B, 5B, and 5D. Twenty-two dominance associations were identified on chromosomes 3A, 3D, 5A, 5B, and 5D. None of the hQTL coincided with dQTL. In Series II, one hQTL was identified on chromosome 1B, with no corresponding dQTL, suggesting an epistatic effect play a role at this locus (Supplementary TableS6; Supplementary Figure S5).

Leaf rust severity showed 55 heterotic associations in Series II on chromosomes 2A, 2B, and 2D, with two mapped to unassigned genomic regions. No significant dominance associations were identified, indicating that the detected heterotic loci are mainly contributed by epistatic interactions (Supplementary Table S7; Supplementary Figure S6). In contrast, no significant associations were detected for yellow rust severity in Series I (Supplementary Figure S6).

## Discussion

While hybrid wheat offers great potential to improve yield and yield stability by exploiting heterosis, its cleistogamous floral architecture poses a major challenge, making the optimization of floral biology essential for achieving efficient cross-pollination. In this study, a total of 294 DH lines derived from 10 biparental populations were used to produce hybrids in seed set trials. These trials enabled the joint assessment of male traits, including visual anther extrusion (VAEX), plant height, heading date, and flowering time, as predictors of seed set. This approach extends previous work conducted in a single biparental population (Zapata et al. 2025) by employing a multi-biparental design and evaluating both the resulting hybrids and their parental lines together. To investigate the genetic basis of heterosis, previous studies in wheat have primarily relied on large, diverse hybrid populations (Jiang et al. 2017; Boeven et al. 2020). While these designs improved mapping resolution compared with biparental mating designs, they often reduced power to detect heterotic loci due to the lower minor allele frequencies and genetic dilution. In contrast, the multi-biparental population used here combines the advantages of biparental mapping including enhanced dominance variance and higher allelic differentiation with greater allelic diversity across crosses. This design can enable a more balanced assessment of both dominance and epistatic effects in wheat hybrids.

### Population-specific relevance of anther extrusion and flowering traits for hybrid seed set in wheat

Reliable indirect traits that can predict hybrid seed set from hybrid production trials are of great value for the practical implementation of hybrid wheat. In Series I, visual anther extrusion (VAEX) contributed to seed set, where VAEX_sum_ and VAEX_mean_ showed significant positive partial correlations with seed set (Table 1), indicating that increased anther extrusion was associated with higher hybrid seed production. Plant height (PH) also exhibited a significant positive partial correlation with seed set, suggesting that taller plants may promote more effective pollen dispersal. This is further supported by the significant correlation found for the height differential between parents (*r* = 0.31, *P* < 0.001), which emphasizes that a positive height gradient between the male and female spikes is essential for maximizing pollen gravity-drop and stigmatic coverage. In contrast, VAEX traits were not significantly associated with seed set in Series II. Instead, heading date (HD), flowering time beginning (FT_begin_), and flowering time end (FTend) emerged as key predictors, with partial correlations of *R* = 0.35, 0.31, and 0.38, respectively.

Selection for high anther extrusion is a common practice in hybrid wheat breeding to ensure sufficient pollen release and seed set (Boeven et al. 2018; Sade et al. 2022; El Hanafi et al. 2022; Garst et al. 2023). However, few studies have used such preselection to reduce the influence of anther extrusion on seed set and thereby reveal the contribution of additional male traits. (Zapata et al. 2025) showed that flowering onset and duration, nicking, and plant height became the most effective predictors of seed set once variation in VAEX was reduced through a light preselection of parents with high anther extrusion. In our study, parental lines also originated from high-VAEX backgrounds; however, the predictive value of VAEX differed between series. In Series I, VAEX remained a significant predictor, suggesting that population-specific genetic or environmental factors enhanced pollen release. In Series II, where flowering time traits were more influential, VAEX was not associated with seed set, underscoring that the relevance of male traits to hybrid seed production is contingent on population context.

Seed set was approximately 2.5-fold higher in Series II than in Series I (Table 1). The slight mismatch between heading (Zadoks 59) and the beginning of flowering time (Zadoks 61) observed in Series I may reflect minor developmental asynchrony and could be associated with the lower hybrid seed set observed in this series. Male plants in Series II were on average 36 cm taller and flowered about nine days later, although their flowering period was three days shorter. However, the increase in male plant height did not translate into higher seed set, as indicated by a near-zero correlation (*R* = –0.01), suggesting that excessive height relative to female plants may not be advantageous. The combination of delayed flowering and a more synchronized flowering period may have enhanced cross-fertilization efficiency in Series II. These findings align with previous work: Zapata et al. (2025) emphasized the importance of males with a later flowering onset for improved seed set, while Schneider et al. (2024) showed that the timing of flowering onset is a critical determinant of hybrid seed production. These insights underscore the need for more research in dynamic phenotyping of reproductive traits, particularly in capturing female receptivity and the synchronization between pollen shedding and stigma receptivity. Recent evidence showing that stigma longevity is not a major limiting factor in hybrid seed production (Millan-Blanquez et al. 2025) further supports the importance of evaluating reproductive processes over time. Future studies should build on such dynamic approaches, as demonstrated by (Schmidt et al. 2025), to improve the predictability of hybrid seed set and develop robust, population-specific selection strategies in wheat hybrid breeding.

### Genetic architecture of seed set reflects population-specific divergence in wheat hybrids

Few studies have investigated the genetic architecture of hybrid seed set. Schneider et al. (2024) detected no significant marker–trait associations, highlighting the trait’s complexity. In contrast, Zapata et al. (2025) identified a marker (AX-158531952) on chromosome 1B within a 7.36 Mb interval (538.78–546.14 Mb) associated with seed set. In the present study, two significant associations were detected on chromosome 1B in Series II, but at distinct genomic positions (~ 8.53 and ~ 249.15 Mb), and additional associations were found on chromosomes 1A and 2A. In Series I, two SNPs (BS00044332_51 at 10,885,844 bp and AX-158547359 at 10,886,513 bp) on chromosome 2B were associated with seed set and located less than 1 kb apart (Supplementary Table S3). Given their close physical proximity, these likely represent a single QTL for seed set on chromosome 2B. This locus lies within the previously reported QTL cluster 2B-I (Cao et al. 2020), which harbors QTLs for three yield components of wheat, such as thousand-kernel weight, kernel number per spike, and spike number per square meter. The overlap suggests that this genomic region may influence multiple yield-related traits, highlighting its potential importance in reproductive success. In addition, two further significant associations were detected at ~ 14.8–15.7 Mb in Series I, located just outside the 2B-I cluster (Cao et al. 2020). These may represent an adjacent or extended locus, potentially reflecting pleiotropic effects on yield components, though direct evidence for pleiotropy in this population requires further validation.

The contrasting QTLs detected between Series I and Series II likely reflect genotype × environment interactions. Seed set is highly sensitive to environmental factors such as temperature, humidity, and pollination dynamics, which influence pollen viability and fertilization success (Boeven et al. 2018; Schneider et al. 2021; El Hanafi et al. 2022; Zapata et al. 2025). Differences in growing conditions and genetic background between series may have altered the expression or detectability of seed set-related loci. These results emphasize the need to evaluate hybrid seed set across multiple genetic and environmental contexts to identify robust genomic regions contributing to seed production.

### Heterosis in wheat hybrids reflects population-specific genetic

Grain yield exhibited moderate mid-parent heterosis (MPH), averaging 13.7% in Series I and 6.1% in Series II, consistent with previous reports of ~ 10% yield advantages in wheat hybrids (Longin et al. 2013; Jiang et al. 2017). The observed better-parent heterosis (BPH) reaching up to 1.1 Mg ha⁻^1^ in Series I and 1.3 Mg ha⁻^1^ in Series II further highlights the competitive potential of these hybrids against elite parental lines. The consistent expression of heterosis across series confirms hybrid vigor in contrasting genetic backgrounds. Heritability estimates for yield heterosis were moderate to high (*H*^2^ = 0.53–0.63; Table 2), indicating substantial genetic control and feasibility of reliable selection. Both series showed negative mean MPH for heading date (−0.8 days), suggesting overall partial dominance toward earlier flowering.

The plant height MPH was significantly higher in Series I (8.4%) than in Series II (0.9%), reflecting greater phenotypic divergence among parental lines. In Series I, the average absolute parental height difference was 6.9 ± 5.2 cm (range 0.1–29.5 cm), whereas in Series II, the contrast was slightly lower and notably narrower (6.0 ± 4.6 cm, range 0.0–21.7 cm). This greater divergence in Series I translated into higher heterosis expression: 99.3% of hybrids showed positive MPH, with a mean hybrid gain of 6.2 cm (range –1.0 to 16.2 cm). In contrast, only 62.7% of hybrids in Series II exhibited positive MPH, and the mean MPH was close to zero (0.7 cm, range –14.8 to 12.3 cm). The higher heterosis in Series I was also evident in the better-parent heterosis, with BPH for plant height reaching 9.7 cm, compared to 8.8 cm in Series II (Supplementary Table S9). The relationship between parental divergence and heterosis also differed between series: Parental height contrast was positively correlated with MPH in Series I (*r* = 0.41), but no such relationship was observed in Series II (*r* = –0.17). Together, these findings indicate that enhanced heterosis in Series I was directly linked to greater parental height divergence, while the more uniform and generally taller parents in Series II limited both the magnitude and consistency of hybrid vigor.

Negative mid-parent heterosis for disease resistance indicates hybrid combinations surpassing parental resistance. In Series I, yellow rust severity was reduced by up to − 1.3 units; in Series II, leaf rust severity reached − 1.8 units (Table 2). Notably, better-parent heterosis (BPH) for YR and LR reached negative values of up to –44.0% (Supplementary Table S9). Despite near-zero average heterosis, the broad range and moderate heritability for leaf rust (*H*^2^ = 0.50) suggest that specific parental interactions enhance resistance. This pattern aligns with prior findings that hybrid performance in wheat is often driven by non-additive genetic effects and the complementation of resistance alleles contributed by both parents (Longin et al. 2013; Beukert et al. 2020), which may confer increased resilience to biotic stresses in specific hybrid combinations where the F₁ outperforms even the most resistant parental lines.

It is important to consider that disease pressure in the field may have influenced grain yield, particularly under natural infection conditions. Differences in resistance levels among hybrids could have contributed to yield variation, potentially confounding the estimation of heterotic effects for grain yield. Therefore, part of the observed heterosis may reflect not only intrinsic yield potential but also differential responses to disease.

### Heterosis in wheat arises from complementary dominance and epistatic effects

Compared with the genome-wide study of Li et al. (2025), which used whole-genome resequencing in three large diverse hybrid panels and highlighted the important role of epistatic interactions in heterosis, our analysis in a multi-parental population revealed a more balanced contribution of both dominance and epistatic effects. This difference may be partly attributable to the markedly distinct minor allele frequency structure observed in our populations (Supplementary Figure S7). This pattern is consistent with the underlying population structure, where parental lines contribute unevenly across multiple interconnected biparental families, resulting in heterogeneous allele representation. Consequently, loci derived from parents involved in fewer crosses may be underrepresented, potentially reducing the power to detect low-frequency effects.

Although a larger number of F_1_ crosses would increase the statistical power for detecting marker–trait associations, particularly for complex traits such as grain yield, the overall level of genetic variation in this study is relatively constrained. The DH lines were derived from a limited set of parental lines, and the testers were drawn from the same genetic background. Within this context, the number of hybrid combinations (~ 300) provides a representative sampling of the available genetic diversity and is sufficient to capture major heterotic effects. Nevertheless, the moderate population size may limit the detection of loci with small effects.

In this study, 28 dominance QTL and 17 heterotic QTL were detected for grain yield in Series I, with five overlapping on chromosome 5B. This pattern suggests that heterosis in wheat arises from complementary mechanisms, where both dominance and epistasis jointly shape hybrid performance depending on the genetic background.

While identifying the parental origin of favorable alleles is highly valuable for breeding applications, this becomes inherently complex for hQTL that do not exhibit significant dominance effects. The phenotypic contribution of a specific allele is strongly dependent on the genetic background and may vary across hybrid combinations. Therefore, assigning a universally “positive” or “negative” effect to alleles from specific parents may oversimplify the underlying genetic architecture. But, the component effects at specific loci, particularly dominance effects, can still be partially interpreted. For example, heterozygosity at a given marker can contribute to heterosis. Notably, the top three high-MPH crosses in Series I all exhibit heterozygous genotypes at the five key MTA loci identified on chromosome 5B, for which genotype information is provided in Supplementary Table S10. Future work may benefit from explicitly modeling background-dependent effects and epistatic interactions to better leverage hQTL information for breeding strategies, such as optimizing parental combinations or stacking complementary alleles.

For traits other than grain yield, the detected heterotic loci were mostly associated with epistatic rather than dominance effects. Interestingly, a heterotic QTL detected on chromosome 4B (~ 30.6 Mb, Supplementary Table S6) for plant height lies within 3 Mb of *Rht-B1* (Liu et al. 2024). This region is well known for the gibberellin-insensitive DELLA mutations underlying semi-dwarf phenotypes and yield improvement (Peng et al. 1999). Similarly, a marker–trait association for heading date on chromosome 2D (~ 31.4 Mb, Supplementary Table S5) lies within 3 Mb of the major flowering time gene *Ppd-D1* (located at 33.9 Mb; Beales et al. 2007). This region is well known for the pseudo-response regulator (PRR) mutations that confer photoperiod insensitivity, providing broad adaptation to diverse environments (Beales et al. 2007). Additional MTAs detected for heading date on chromosome 2D (~ 31.4 Mb), six on chromosome 4B (~ 2.3–6.9 Mb), one on chromosome 6D (~ 25.9 Mb), and one on chromosome 7 A (~ 114.6 Mb) fall within QTL-rich clusters for thousand-kernel weight (TKW), kernel number per spike (KNS), and spike number per square meter (SN) previously reported by Cao et al. (2020).

## Conclusion

Our results demonstrate that population-specific strategies are required in hybrid wheat breeding, as the importance of male trait such as anther extrusion, flowering time, and plant height for seed set depends strongly on genetic background and environmental conditions. Rather than relying on single morphological predictors, breeding efforts should prioritize flowering synchronization and dynamic phenotyping of reproductive traits. Multi-environment testing is essential to capture strong genotype × environment interactions that influence seed set and heterosis. In addition, genomic selection models should explicitly account for dominance and epistatic effects, which jointly contribute to hybrid performance. Finally, exploiting key genomic regions associated with heterosis and yield stability can enhance both hybrid seed production efficiency and agronomic resilience.

## Supplementary Information

Below is the link to the electronic supplementary material.

Supplementary file1 (XLSX 387 KB)

Supplementary file2 (DOCX 2156 KB)

## Data Availability

Phenotypic data, including raw and processed data (BLUEs and BLUPs) from seed set and hybrid performance trials, together with R scripts for data processing and statistical analyses, are available in the e!DAL-PGP Repository (Arend et al. 2016) and can be accessed here (Zapata 2026).
