# Supplementary material for: Association mapping of hybrid seed set and heterosis in a central European multi-parental wheat population
Source: Theor Appl Genet. 2026 Apr 28;139(5):140. doi: 10.1007/s00122-026-05247-0 (PMC13124887; doi:10.1007/s00122-026-05247-0)
Supplement: Supplementary file 2 — Supplementary file2 (DOCX 2156 KB) [file 122_2026_5247_MOESM2_ESM.docx]

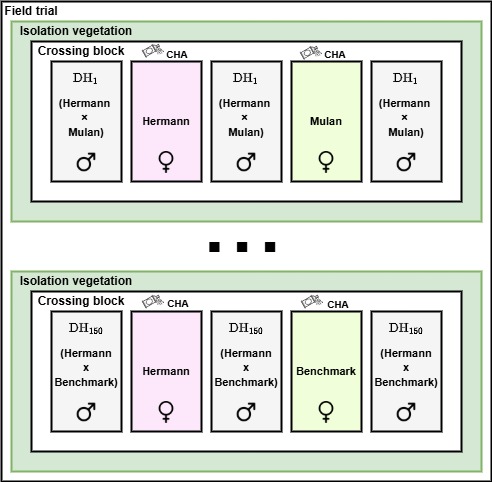


**Supplementary Figure S1.** Schematic representation of the crossing block design used for hybrid seed production. Each doubled haploid (DH) line (♂), derived from a biparental cross (indicated in parentheses), was used as a pollen donor and crossed with its two corresponding parental lines, which served as female testers (♀). Female plants were emasculated using chemical hybridizing agents (CHAs) and arranged in isolated crossing blocks. Each plot was surrounded by a buffer crop to prevent cross-pollination between different DH lines.


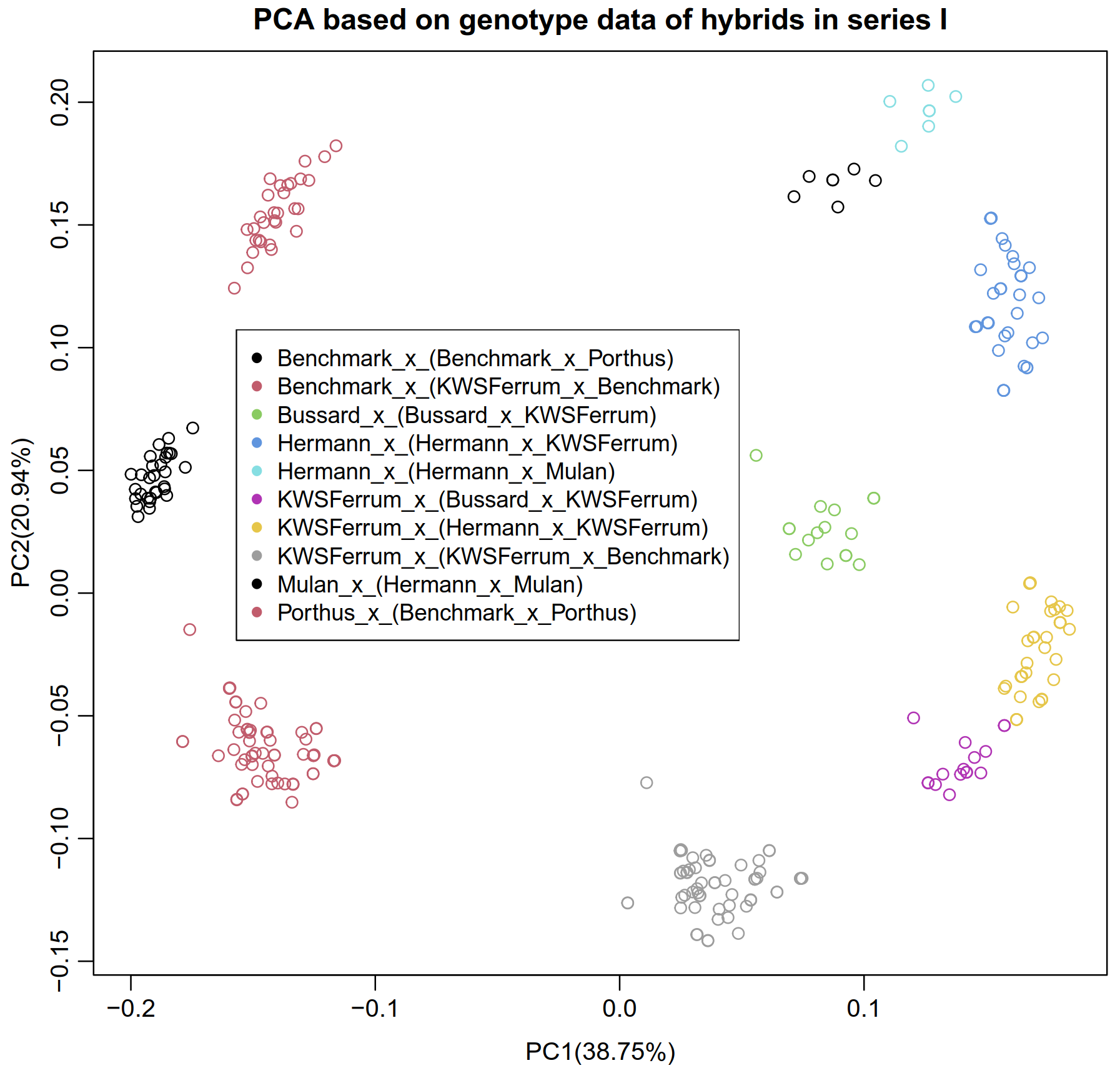


**Supplementary** Figure S2. Principal component analysis (PCA) of F_1_ hybrids in Series I based on genotype data. Each point represents an individual F_1_ hybrid, colored according to its parental cross. PC1 and PC2 explain 38.75% and 20.94% of the total genetic variance, respectively.


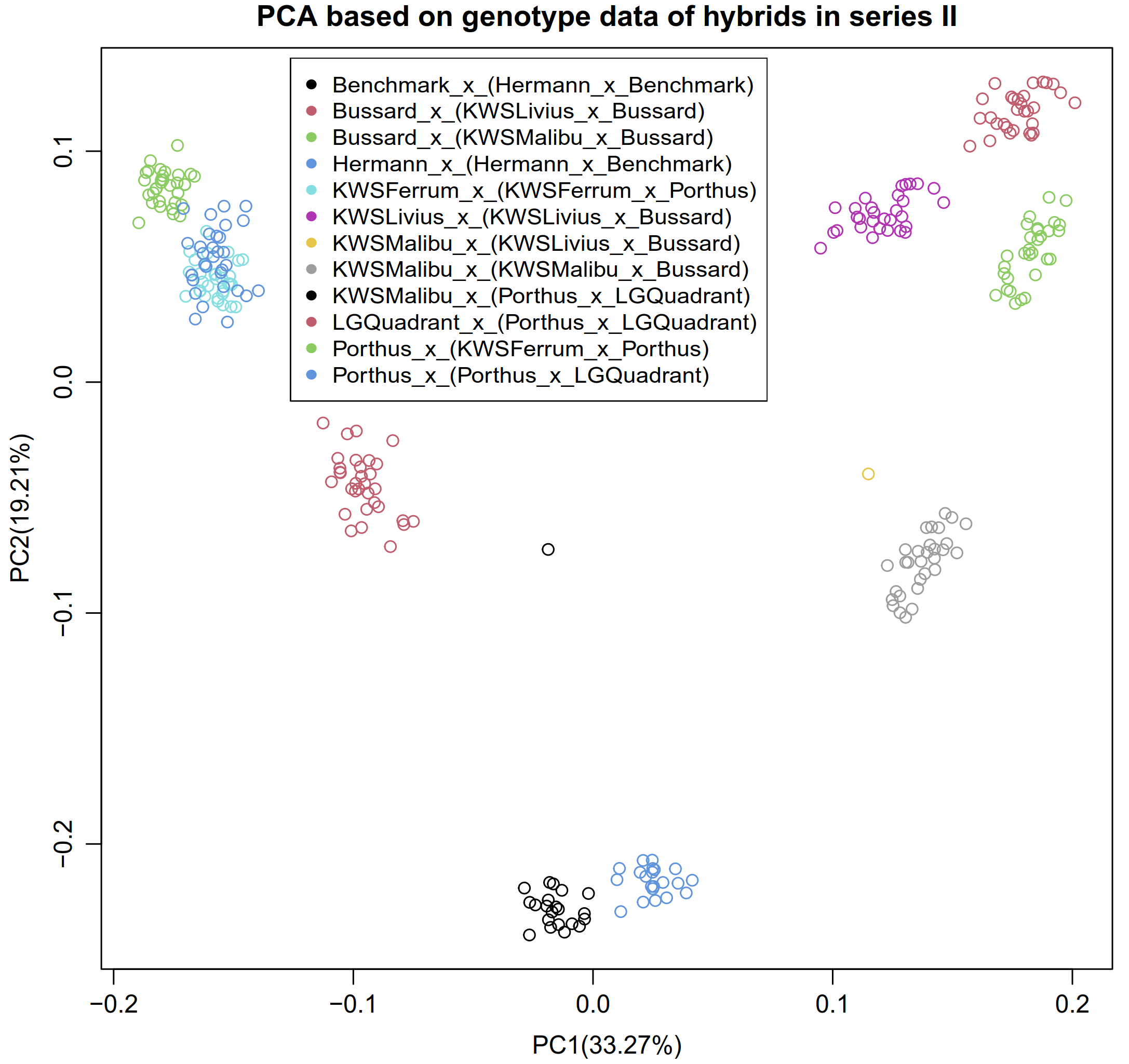


**Supplementary** Figure S3. Principal component analysis (PCA) of F_1_ hybrids in Series II based on genotype data. Each point represents an individual F_1_ hybrid, colored according to its parental cross. PC1 and PC2 explain 33.27% and 19.21% of the total genetic variance, respectively.


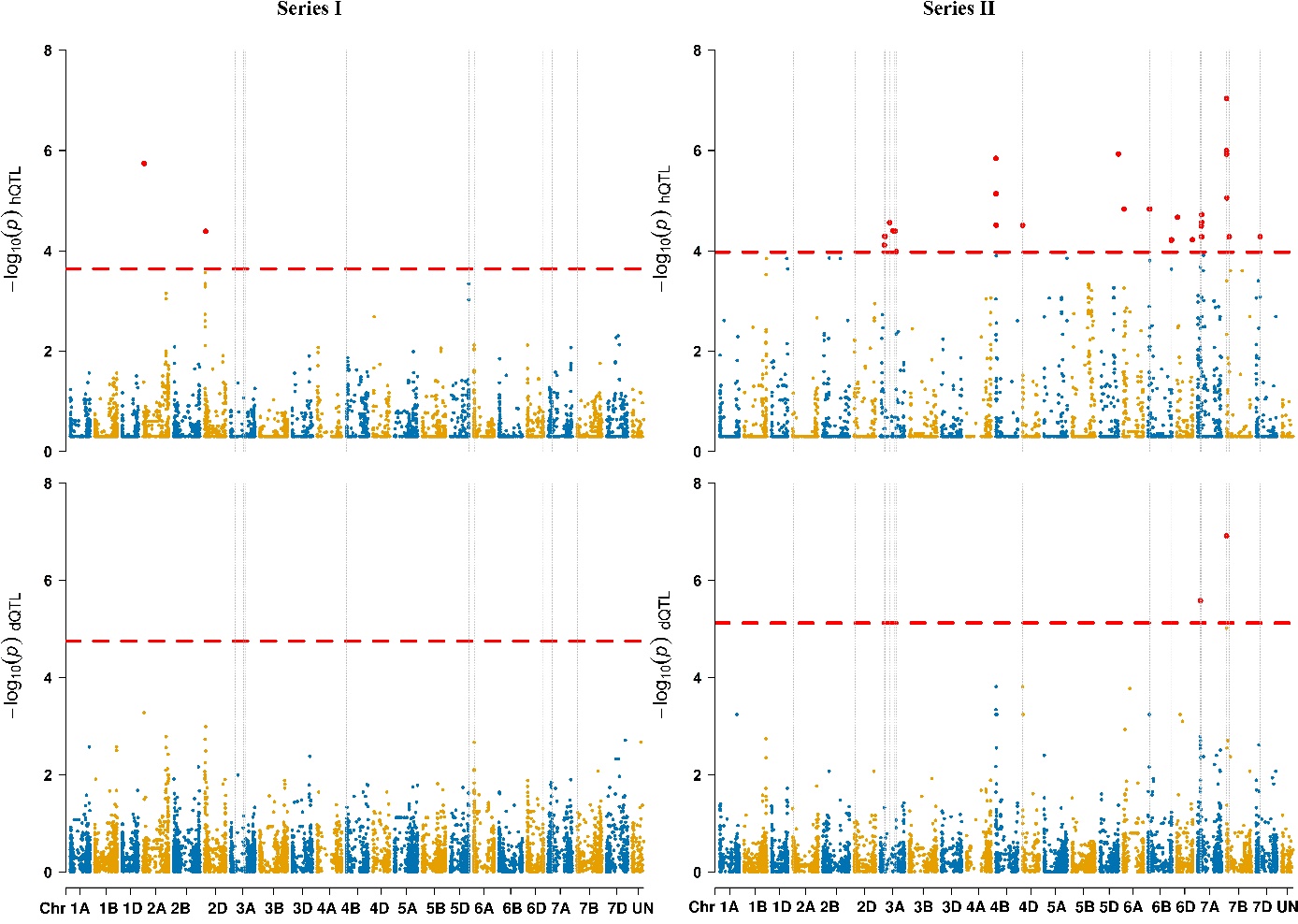


**Supplementary Figure S4.** Manhattan plots of heterotic QTL (hQTL) and dominance QTL (dQTL) analyses for heading date (HD) in Series I (SI) and Series II (SII). The red dashed line indicates the significance threshold (α = 0.05) derived from 500 permutation tests. Significant marker–trait associations exceeding this threshold are highlighted. The scale of p-values corresponds to −log_10_(p -value), while positions (Mb) of 14,634 high-quality SNPs on the chromosome sequence maps are according to RefSeq 2.1 of Chinese Spring (Zhu *et al.*, 2021).


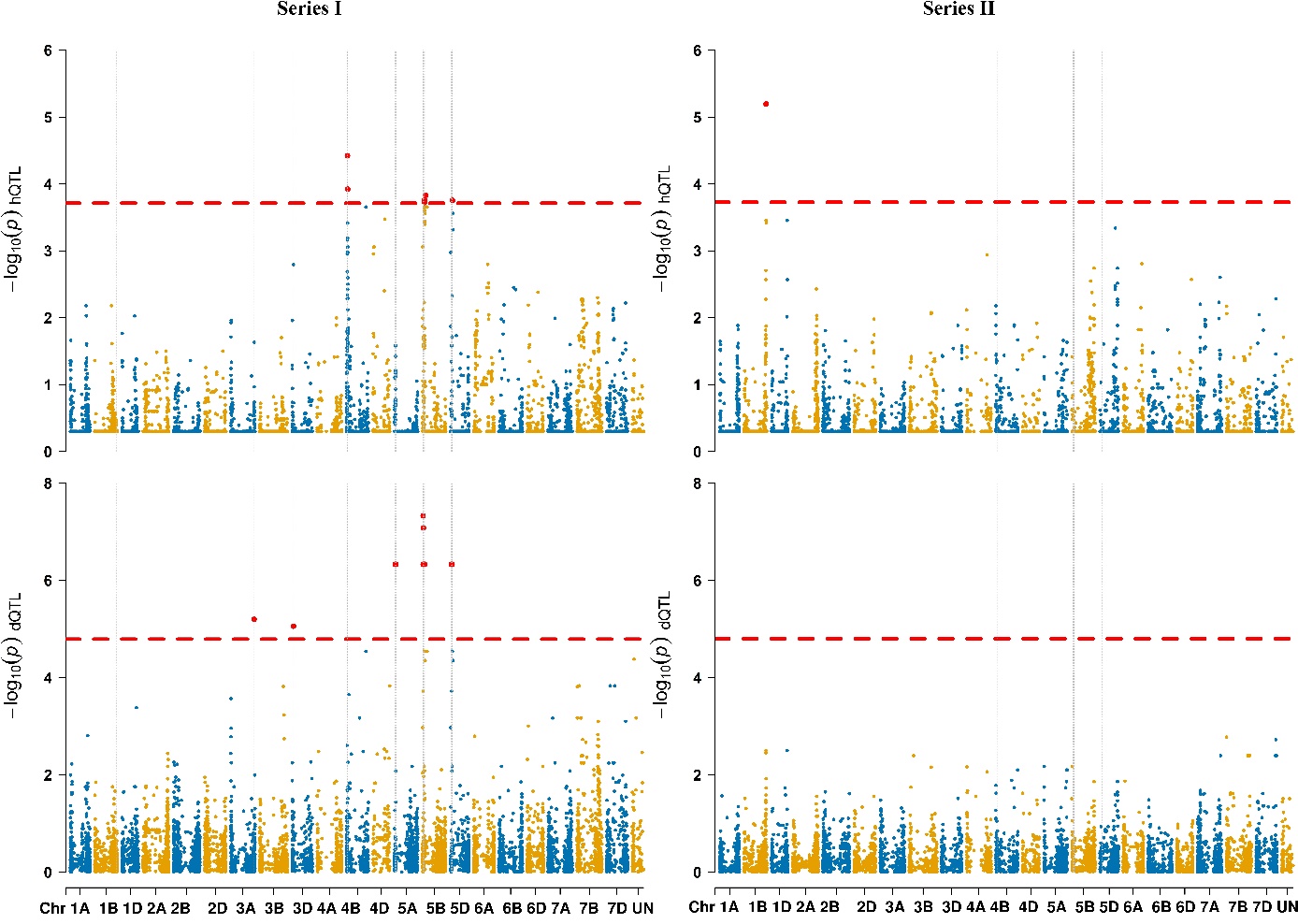


**Supplementary Figure S5.** Manhattan plots of heterotic QTL (hQTL) and dominance QTL (dQTL) analyses for plant height (PH) in Series I (SI) and Series II (SII). The red dashed line indicates the significance threshold (α = 0.05) derived from 500 permutation tests. Significant marker–trait associations exceeding this threshold are highlighted. The scale of p-values corresponds to −log_10_(p -value), while positions (Mb) of 14,634 high-quality SNPs on the chromosome sequence maps are according to RefSeq 2.1 of Chinese Spring (Zhu *et al.*, 2021).


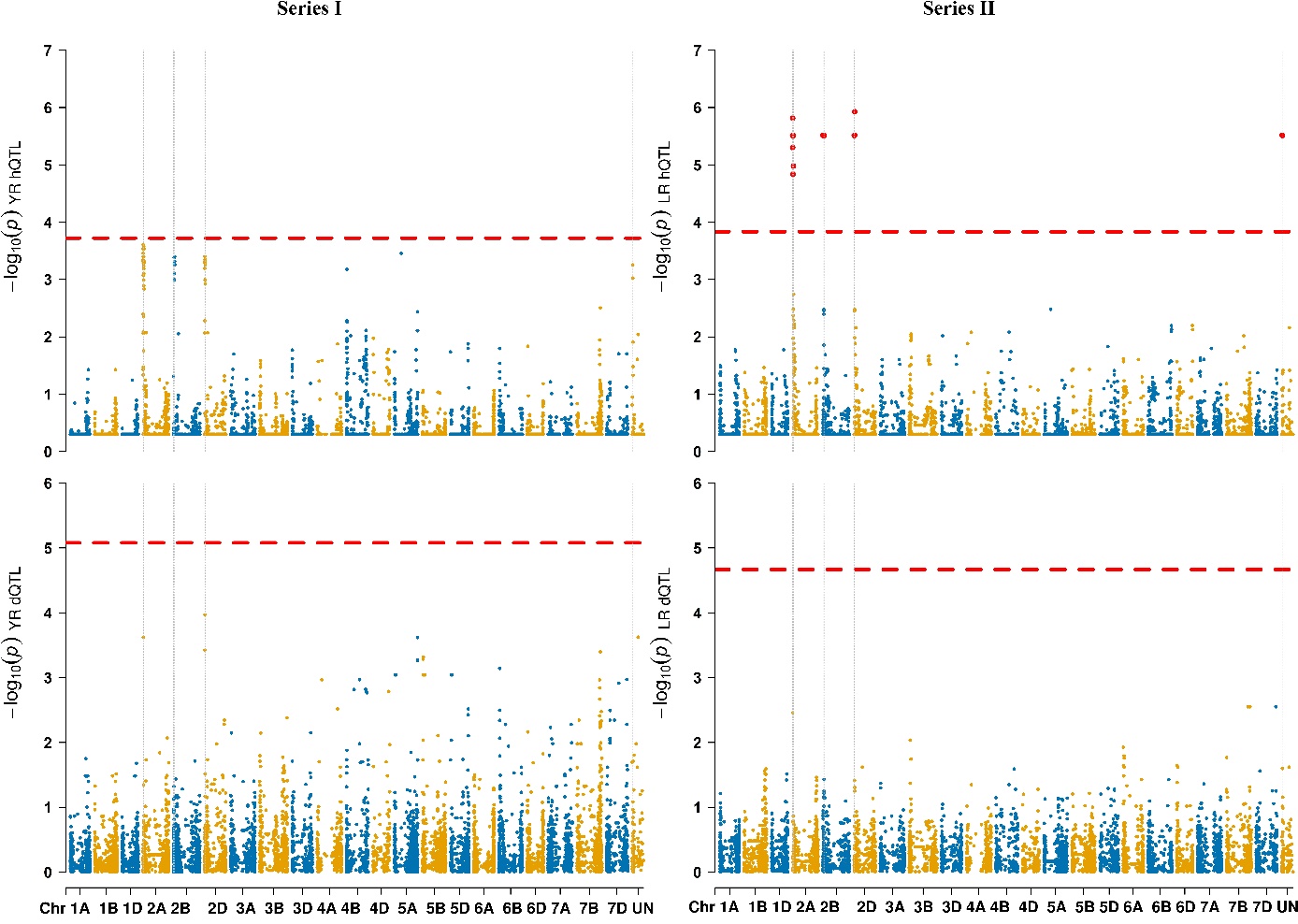


**Supplementary Figure S6.** Manhattan plots of heterotic QTL (hQTL) and dominance QTL (dQTL) analyses for yellow rust (YR) in Series I (SI) and leaf rust (LR) in Series II (SII). The red dashed line indicates the significance threshold (α = 0.05) derived from 500 permutation tests. Significant marker–trait associations exceeding this threshold are highlighted. The scale of p-values corresponds to −log_10_(p -value), while positions (Mb) of 14,634 high-quality SNPs on the chromosome sequence maps are according to RefSeq 2.1 of Chinese Spring (Zhu *et al.*, 2021).


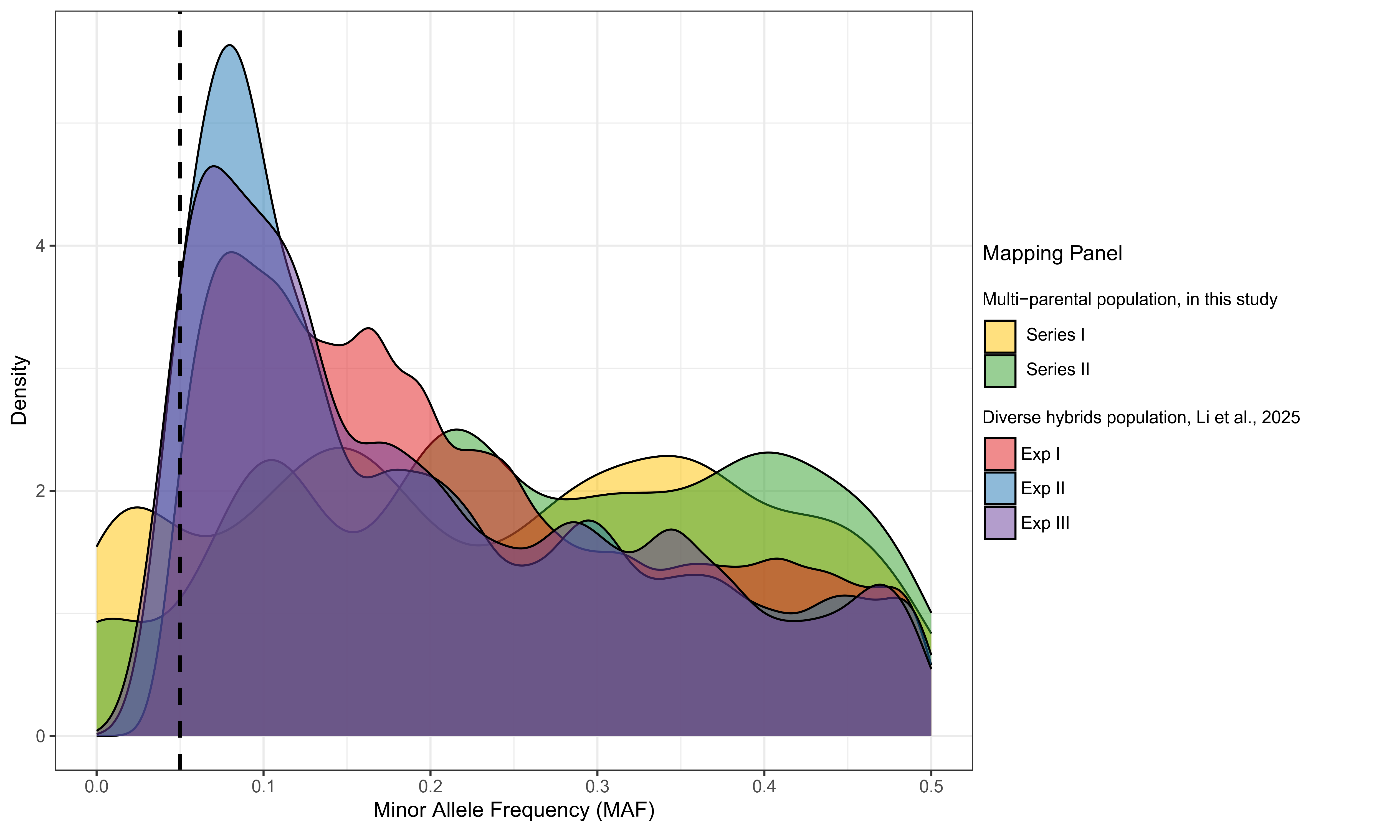


**Supplementary Figure S7.** Comparative minor allele frequency (MAF) distribution in current multi-biparental mapping populations (Series I and Series II) versus external large diverse hybrid panels (Li et al., 2025). The dashed black line indicates the critical low-MAF threshold (0.05). The comparison of these distributions illustrates the differences in allele frequency structure between the population designs used, contextualizing the findings relative to large genomic studies (Li et al., 2025).
